# Real-world use of brexpiprazole during inpatient treatment for schizophrenia: continuation, discontinuation, and concomitant psychotropics

**DOI:** 10.3389/fpsyt.2026.1829496

**Published:** 2026-05-05

**Authors:** Yuki Noriyama, Shintaro Araki, Ryohei Takada, Hiroaki Fukui, Yuya Honda, Kazuki Okumura, Yuki Nishi, Minobu Ikehara, Takashi Okada

**Affiliations:** Department of Psychiatry, Nara Medical University School of Medicine, Kashihara, Japan

**Keywords:** brexpiprazole, concomitant medications, discontinuation, inpatient, real-world evidence, schizophrenia, treatment continuation

## Abstract

**Introduction:**

In the treatment of schizophrenia, antipsychotics used during acute inpatient care must control acute symptoms while remaining sufficiently tolerable to support treatment beyond the acute phase. Brexpiprazole, a serotonin-dopamine activity modulator may be one such option; however, its real-world use and short-term continuation in acute inpatient settings remain insufficiently characterized.

**Methods:**

We conducted a retrospective observational study of inpatients with DSM-5 schizophrenia treated with brexpiprazole at a university hospital in Japan between June 2018 and July 2024. The index date (week 0) was defined as the date of brexpiprazole initiation during the index hospitalization. The primary outcome was brexpiprazole continuation at week 8. We compared baseline demographic and treatment-related variables between the continuation and discontinuation groups and summarized reasons for discontinuation from electronic medical records. As a secondary exploratory analysis, we examined longitudinal changes in Clinical Global Impressions–Severity scale (CGI-S) and Brief Psychiatric Rating Scale (BPRS) total scores (weeks 0/4/8) in the continuation group using a linear mixed-effects model including time, concomitant psychotropic medication status, and their interaction.

**Results:**

Sixty-seven patients were included. Baseline illness severity was substantial (median CGI-S 5.0 [IQR 5.0–6.0]; mean BPRS total 58.5 ± 9.6). Concomitant psychotropic medications were common. Thirty-six patients continued brexpiprazole to week 8 (53.7%). In unadjusted exploratory comparisons, continuation was associated with the female sex (p = 0.036), lower prior chlorpromazine-equivalent dose (p = 0.015), and shorter duration of untreated psychosis (p = 0.003), with a trend toward shorter duration since onset (p = 0.073). The most frequent reason for discontinuation was adverse events (n = 10, 32.3%), most commonly akathisia (n = 6), followed by insufficient efficacy (n = 9, 29.0%) and patient preference/refusal (n = 7, 22.6%). In exploratory mixed-effects analyses within the continuation group, CGI-S and BPRS total scores decreased over time, with significant group-by-time interactions by concomitant medication status. However, between-group differences should be interpreted cautiously.

**Discussion:**

This study describes 8-week continuation and reasons for discontinuation of brexpiprazole in acute inpatient schizophrenia care. Given the retrospective single-center design and potential selection/information bias and unmeasured confounding, further studies are warranted to clarify its clinical positioning in real-world practice.

## 1 Introduction

Schizophrenia is a serious psychiatric disorder, affecting approximately 1% of the global population (1). It is characterized by positive symptoms (e.g., hallucinations and delusions), negative symptoms (e.g., blunted affect and avolition), and cognitive impairments. Antipsychotic pharmacotherapy is the mainstay of treatment, with symptom remission and recovery of social functioning as the key goals. In psychiatric emergency care, clinicians must manage psychomotor agitation rapidly and safely. Conversely, when planning for maintenance treatment, clinicians often prefer agents with low sedative properties, a lower risk of extrapyramidal and anticholinergic adverse effects, and potential benefits for cognitive functioning (2–4). Moreover, in routine clinical practice, clinicians must select antipsychotic treatments while considering multiple factors, including illness severity, the need for concomitant medications, patients’ social circumstances, and the inpatient ward environment (5, 6). Accordingly, effectively and safely managing acute symptoms while anticipating the maintenance phase requires consideration of influence of these diverse factors. In this context, clarifying real-world prescribing patterns and treatment continuation in acute-care settings remains an important clinical challenge.

Brexpiprazole is an antipsychotic classified as a serotonin-dopamine activity modulator. It acts as a partial agonist at dopamine D2 and serotonin 5-HT1A receptors and as an antagonist at 5-HT2A receptors (7). Although it is pharmacologically similar to aripiprazole, brexpiprazole has lower intrinsic activity at D2 receptors and relatively higher affinity for 5-HT2A receptors than aripiprazole (8, 9). Because 5-HT2A antagonism may increase dopaminergic transmission in the nigrostriatal pathway, this receptor profile may contribute to a more moderate dopaminergic profile and favorable tolerability (10). Das et al. reported that brexpiprazole has moderate-to-low affinity for histamine H1 and muscarinic M1 receptors, which may account for its lower risk of sedation, metabolic disturbances, and cognitive effects (11). Consistently, recent large network meta-analyses have reported lower rates of such adverse effects with brexpiprazole (12, 13). Accordingly, when clinicians prioritize treatment persistence and tolerability beyond the acute phase, brexpiprazole may represent a therapeutic option.

Brexpiprazole has been shown to significantly improve anxiety-related Positive and Negative Syndrome Scale (PANSS) items compared with placebo, which may, in turn, help ameliorate psychomotor agitation in patients experiencing acute episodes (14). An international multicenter randomized controlled trial by Correll et al. demonstrated symptomatic improvement with brexpiprazole and its good tolerability in patients with acute schizophrenia, with a phase III trial conducted in Japan reporting similar results (15, 16). However, most of this evidence derives from controlled research settings with predefined eligibility criteria, and real-world data on prescribing patterns and short-term treatment continuation in acute inpatient care remain limited.

This study aimed to investigate the real-world use of brexpiprazole among patients hospitalized with acute schizophrenia at a university hospital in Japan. Specifically, the primary outcome was 8-week treatment continuation from the initiation date of brexpiprazole during hospitalization (index date; week 0), with the reasons for discontinuation, including adverse events, retrospectively described. In addition, we explored clinical factors associated with treatment continuation and examined trajectories of clinician-rated symptom scales as secondary outcomes.

## 2 Materials and methods

### 2.1 Study design

This retrospective observational study was approved by the Nara Medical University Ethics Committee (approval number: 2246) in June 2019 and conducted in accordance with the ethical principles of the Declaration of Helsinki. The study used routinely collected medical record data under institutional ethical oversight. An opt-out procedure was implemented via public disclosure of study information on the department website. This retrospective study was designed and reported according to the STROBE statement for observational studies.

### 2.2 Participants

This retrospective observational study included patients hospitalized in the Department of Psychiatry at Nara Medical University Hospital in Japan between June 1, 2018, and July 31, 2024. The patients met the Diagnostic and Statistical Manual of Mental Disorders, Fifth Edition (DSM-5) criteria for schizophrenia and were prescribed brexpiprazole at the discretion of the treating psychiatrist. To reduce selection bias, we included all consecutive inpatients who met the eligibility criteria during the study period. To minimize information bias, clinical data were extracted from electronic medical records using a predefined template. For this study, the index date (week 0) was defined as the date on which brexpiprazole was initiated during the index hospitalization; for patients who had been taking brexpiprazole prior to admission, the admission date was used as week 0. Patients were excluded if baseline information required for the analyses was insufficient; if they died, were transferred to another facility, or were discharged within 8 weeks of the index date (week 0); or if brexpiprazole was prescribed as an adjunctive (augmentation) agent rather than as the primary antipsychotic treatment. Those patients who continued brexpiprazole for 8 weeks were classified into the continuation group, whereas the others were classified into the discontinuation group.

### 2.3 Measures

From the hospital’s electronic medical records, we extracted baseline patient characteristics at index date (week 0), including age, sex, age at onset, duration of untreated psychosis (DUP), duration since onset (time from onset to index date), and prior chlorpromazine-equivalent (CPZ-eq) dose. We also recorded treatment-related variables during the 8-week observation period, including the brexpiprazole dose at steady state, the presence of concomitant psychotropic medications at index date or initiated within 8 weeks thereafter (excluding medications prescribed for short-term, as-needed use) and use of seclusion or restraint. In addition, we classified brexpiprazole treatment status as follows: continued from prior to admission (ongoing use before admission and continued during hospitalization), newly initiated during hospitalization (no use prior to admission), or switched from another antipsychotic during hospitalization (including cross-titration). These treatment-status groups represented clinically distinct patient populations but were analyzed together.

The primary outcome was brexpiprazole continuation at week 8 from index date. This definition was intended to capture short-term treatment persistence under inpatient clinical conditions. Secondary outcomes were as follows: (1) baseline demographic and treatment characteristics by continuation status; within the continuation group, we also explored subgroup differences according to concomitant psychotropic medication use; (2) reasons for discontinuation, including adverse events; and (3) exploratory trajectories of the Clinical Global Impressions–Severity scale (CGI-S) and the Brief Psychiatric Rating Scale (BPRS) at week 0, week 4, and week 8 (and changes from baseline) based on routine clinical ratings (17–20).

### 2.4 Statistical analysis

Continuous variables with approximately normal distribution are expressed as mean ± standard deviation and were compared between groups using Welch’s t-test. Non-normally distributed continuous variables were summarized as median (interquartile range, IQR) and compared using the Mann–Whitney U test. Dichotomous variables are expressed as n (%) and were analyzed using Fisher’s exact test.

Within the continuation group, longitudinal changes in CGI-S and BPRS total scores at weeks 0, 4, and 8 were analyzed using a linear mixed-effects model with fixed effects for time, concomitant psychotropic medication status, and their interaction and a participant-specific random intercept; model parameters were estimated by restricted maximum likelihood (REML). CGI-S and BPRS data were available for all continuation-group patients at 0, 4, and 8 weeks. *Post hoc* pairwise comparisons across time points were adjusted using Tukey’s method; between-group comparisons at each time point were adjusted using the Holm–Šídák method. All analyses were performed using GraphPad Prism version 9 (GraphPad Software, San Diego, CA, USA). Two-sided p-values <0.05 were considered statistically significant, and findings from symptom-rating analyses were interpreted as exploratory.

## 3 Results

### 3.1 Patient characteristics

During the study period, 79 patients with schizophrenia received brexpiprazole. Twelve patients were excluded because baseline information was insufficient (n = 5); they died, were transferred to another facility, or were discharged within 8 weeks of the index date (n = 1 each); or brexpiprazole was prescribed as adjunctive (augmentation) therapy rather than as the primary antipsychotic (n = 4). In total, 67 patients were included in the analysis. Baseline demographic and treatment characteristics are presented in **Table 1**. The median prior CPZ-eq dose was 600.0 (IQR 0–800.0) mg, the median CGI-S score was 5.0 (IQR 5.0–6.0), and the mean BPRS total score was 58.5 ± 9.6, suggesting that the sample included patients with relatively severe illness. The inclusion of 0 mg in the interquartile range of the prior CPZ-equivalent dose reflects the presence of patients in whom brexpiprazole was newly initiated during hospitalization and who did not receive antipsychotic treatment before the index date. The median duration since onset was 13 years, suggesting that the sample included patients with a longer-standing course of schizophrenia. The median brexpiprazole dose was 2.0 (IQR 2.0–2.0) mg; in most cases, the dose was titrated promptly to 2 mg in accordance with the Japanese package insert. Although the approved maximum dose in Japan is 2 mg/day, two patients in the continuation group received 4 mg/day and one received 3 mg/day with informed consent for off-label use. Overall, 49 patients (73.1%) received concomitant psychotropic medications at index date or initiated within 8 weeks thereafter.

**Table 1:** Baseline characteristics of the study population stratified by brexpiprazole continuation status.

Variable	Total (N=67)	Brexpiprazole continuation group (N=36)	Brexpiprazole discontinuation group (N=31)	p-value
Sex (female, %)	33 (49.2%)	22 (61.1%)	11 (35.5%)	0.036*
Age (years)	40.7 ± 15.1	38.4 ± 16.7	42.6 ± 14.4	0.284
Age at onset (years)	25.2 ± 9.8	25.8 ± 9.3	24.7 ± 9.4	0.634
Duration since onset (years)	13.0 (5.0–25.0)	9.5 (1.1–19.0)	15.0 (6.0–26.0)	0.073^†^
DUP (years)	1.0 (0.5–2.0)	1.0 (0.3–2.0)	1.5(1.0–3.0)	0.003**
Prior CPZ-eq dose (mg)	600.0 (0–800.0)	400.0 (0–750.0)	750.0 (250.0–800.0)	0.015*
Brexpiprazole dose (mg)	2.0 (2.0–2.0)	2.0 (2.0–2.0)	2.0 (2.0–2.0)	0.158
Baseline CGI-S score	5.0 (5.0–6.0)	5.0 (5.0–6.0)	5.0 (5.0–6.0)	0.773
Baseline BPRS score	58.5 ± 9.6	60.3 ± 8.4	58.0 ± 10.3	0.342
Seclusion and restraint (present, %)	30 (44.8%)	13 (36.1%)	17 (54.8%)	0.146
Presence of concomitant psychotropic medications (present, %)	49(73.1%)	24(66.7%)	25(80.7%)	0.271
Distribution of brexpiprazole treatment status				0.311
Continued from prior to admission	5 (7.5%)	2 (5.6%)	3 (9.7%)	–
Newly initiated during hospitalization	19 (28.3%)	13 (36.1%)	6 (19.4%)	–
Switched from another antipsychotic during hospitalization	43 (64.2%)	21 (58.3%)	22 (71.0%)	–

Continuous variables are presented as mean ± standard deviation or median (interquartile range), and categorical variables as n (%). Comparisons between the brexpiprazole continuation and brexpiprazole discontinuation groups are indicated by p-values, calculated using Welch’s t-test or the Mann–Whitney U test for continuous variables and Fisher’s exact test for categorical variables. For 2×3 tables, the Freeman–Halton extension of Fisher’s exact test was used. P values are unadjusted and are provided for exploratory descriptive comparisons only.

SD, standard deviation; DUP, duration of untreated psychosis; CPZ-eq dose, chlorpromazine-equivalent dose; CGI-S, Clinical Global Impressions–Severity scale; BPRS, Brief Psychiatric Rating Scale. ^†^0.05<p<0.1; *p<0.05; **p<0.01.

### 3.2 Baseline demographic and treatment characteristics by continuation status

Of the 67 patients, 36 continued brexpiprazole for 8 weeks (continuation group) and 31 discontinued earlier (discontinuation group), resulting in an 8-week continuation rate of 53.7%. **Table 1** summarizes baseline demographic and treatment characteristics by continuation status. In terms of baseline demographic characteristics, compared with the discontinuation group, the continuation group included a higher proportion of female patients (p = 0.036), had a lower prior CPZ-eq dose (p = 0.015), and showed a shorter DUP (p = 0.003). Duration since onset tended to be shorter in the continuation group (p = 0.073). Age, age at onset, baseline CGI-S score, and baseline BPRS total score did not differ significantly between the groups. BPRS item scores are shown in **Supplementary Table S1**. In terms of treatment characteristics, brexpiprazole dose, presence of concomitant psychotropic medications at index date or initiated within 8 weeks thereafter, use of seclusion or restraint and the distribution of brexpiprazole treatment status did not differ significantly between groups. These comparisons were unadjusted and thus should be interpreted as exploratory descriptive findings rather than evidence of independent associations.

Within the continuation group, we further examined concomitant psychotropic medication use as an exploratory subgroup analysis. Twelve patients did not receive concomitant psychotropics while 24 received concomitant psychotropics (excluding short-term, as-needed medications). The most commonly used concomitant drugs were bromazepam (n = 7) and olanzapine (n = 5); the full list is provided in **Supplementary Table S2**. Baseline characteristics of these subgroups are shown in **Table 2**. The prior CPZ-eq dose was lower in the non-concomitant group (p = 0.031), whereas other baseline variables did not differ significantly.

**Table 2:** Comparison of baseline characteristics between the non-concomitant and concomitant medication groups in the brexpiprazole continuation group.

Variable	Non-concomitant medication group (N=12)	Concomitant medication group (N=24)	p-value
Sex (female, %)	7 (58.3%)	15 (62.5%)	0.809
Age (years)	34.8 ± 17.2	40.3 ± 16.5	0.366
Age at onset (years)	27.1 ± 9.1	25.1 ± 9.51	0.550
Duration since onset (years)	5.5 (1.0–10.8)	13.5 (1.6–23.8)	0.190
DUP (years)	1.0 (0.3–2.0)	0.8 (0.3–2.0)	0.915
Prior CPZ-eq dose (mg)	0 (0–400.0)	466.5 (50.0–800.0)	0.031*
Brexpiprazole dose (mg)	2.0 (2.0–2.0)	2.0 (2.0–2.0)	0.897
Baseline CGI-S score	5.0 (5.0–6.0)	5.5 (5.0–6.0)	0.482
Baseline BPRS score	58.8 ± 6.8	61.0 ± 9.2	0.322
Seclusion and restraint (present, %)	6 (50.0%)	12 (50.0%)	>0.999
Distribution of brexpiprazole treatment status			0.007**
Continued from prior to admission	1 (8.3%)	1 (4.2%)	–
Newly initiated during hospitalization	8 (66.7%)	5 (20.8%)	–
Switched from another antipsychotic during hospitalization	3 (25.0%)	18 (75.0%)	–

Continuous variables are presented as mean ± standard deviation or median (interquartile range), and categorical variables as n (%). Comparisons between the non-concomitant and concomitant medication groups are indicated by p-values, calculated using Welch’s t-test or the Mann–Whitney U test for continuous variables and Fisher’s exact test for categorical variables, as appropriate. For 2×3 tables, the Freeman–Halton extension of Fisher’s exact test was used.

SD, standard deviation; DUP, duration of untreated psychosis; CPZ-eq dose, chlorpromazine-equivalent dose; CGI-S, Clinical Global Impressions–Severity scale; BPRS, Brief Psychiatric Rating Scale. *p<0.05; **p<0.01.

### 3.3 Reasons for discontinuation

In the discontinuation group, the main reasons for treatment discontinuation were adverse events in 10 patients (32.3%), insufficient efficacy in nine (29.0%), patient preference or refusal in seven (22.6%), and unknown reasons in five (16.1%). Reported adverse events included akathisia (n = 6), sleep disturbance (n = 1), extrapyramidal symptoms (n = 1), nausea (n = 1), and fatigue (n = 1), all of which were mild in severity. Only the case of fatigue was considered related to sedation.

### 3.4 Exploratory trajectories of CGI-S and BPRS in the continuation group

**Figure 1:**
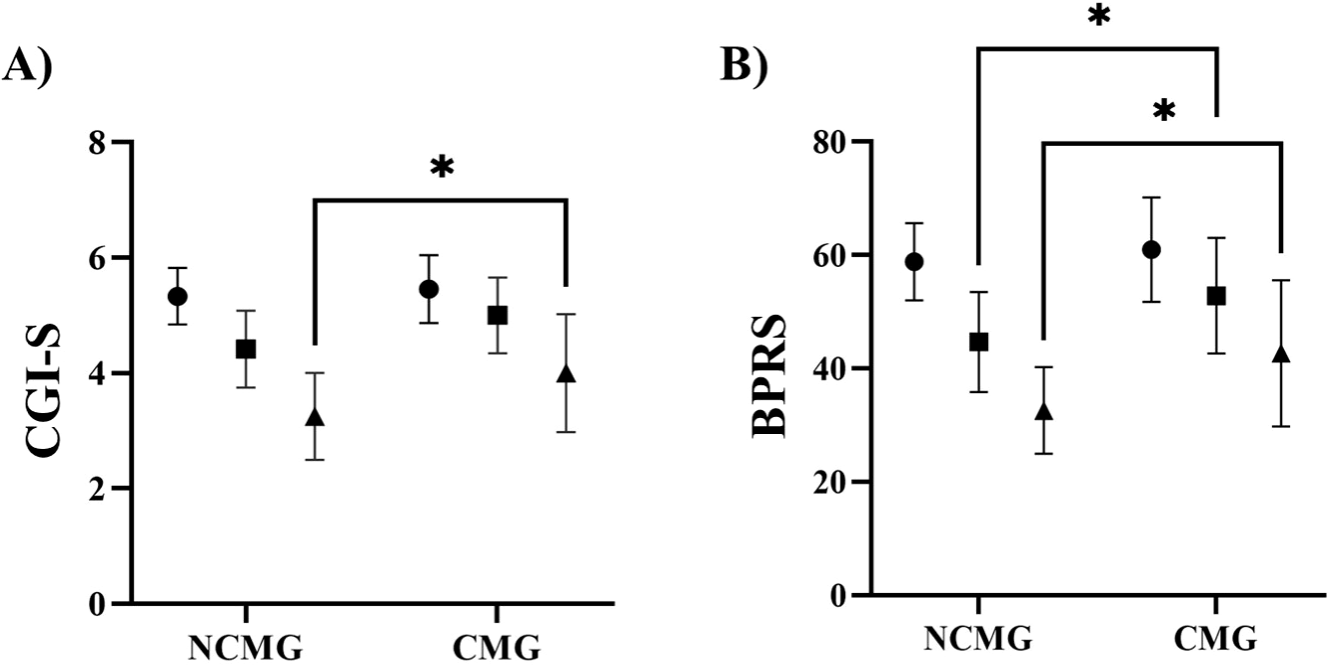
Exploratory longitudinal changes in clinical rating scales (comparison by concomitant medication status). **(A)** CGI-S. **(B)** BPRS total score. Each point represents the mean and error bars represent the standard deviation. ● denotes week 0, ■ week 4, and ▲ week 8. Trajectories were analyzed using a linear mixed-effects model with fixed effects for group (non-concomitant vs concomitant), time (categorical), group-by-time interaction, and a participant-specific random intercept. Model parameters were estimated by REML, restricted maximum likelihood. Post hoc comparisons of time points within each group were Tukey-adjusted, and between-group comparisons at each time point were Holm–Šídák-adjusted. NCMG, Non-Concomitant Medication Group; CMG, Concomitant Medication Group; CGI-S, Clinical Global Impressions–Severity scale; BPRS, Brief Psychiatric Rating Scale; *Adjusted p<0.05.

These exploratory longitudinal analyses were restricted to the continuation group because week-8 ratings were not available for patients who discontinued brexpiprazole earlier. Exploratory longitudinal changes in CGI-S and BPRS total scores at weeks 0, 4, and 8 were evaluated using a linear mixed-effects model with fixed effects for group (non-concomitant vs concomitant), time (categorical), group-by-time interaction, and a participant-specific random intercept, estimated by REML, using all available observations without imputation (**Figure 1**). For CGI-S, the model showed significant effects of group [F(1,34) = 5.08, p = 0.031], time [F(2,68) = 97.4, p < 0.001], and a group-by-time interaction [F(2,68) = 3.20, p = 0.047]. Tukey-adjusted *post hoc* tests indicated significant within-group decreases across all time-point comparisons in both groups. Between-group comparisons at each time point (Holm–Šídák adjusted) showed no difference at baseline (adjusted p = 0.634) and week 4 (adjusted p = 0.055), whereas the non-concomitant group had lower CGI-S scores at week 8 (adjusted p = 0.015).

For BPRS total scores, the model showed significant effects of group [F(1,34) = 4.81, p = 0.035], time [F(2,68) = 114.9, p < 0.001], and a group-by-time interaction [F(2,68) = 4.00, p = 0.023]. Tukey-adjusted *post hoc* tests indicated significant within-group decreases across all time-point comparisons in both groups. Between-group comparisons at each time point (Holm–Šídák adjusted) showed no difference at baseline (adjusted p = 0.548), whereas the non-concomitant group had lower BPRS total scores at week 4 (adjusted p = 0.045) and week 8 (adjusted p = 0.016). These subgroup findings should be interpreted with substantial caution because concomitant psychotropic medication status was not randomized and likely reflected underlying illness severity, treatment complexity, and clinician decision-making, including medications introduced during follow-up. Therefore, these results did not allow the causal or clinically direct interpretation of the effects of concomitant medications themselves.

## 4 Discussion

This retrospective study examined the real-world use of brexpiprazole in patients hospitalized for acute schizophrenia in the psychiatry department of a university hospital, focusing primarily on 8-week treatment continuation and reasons for discontinuation, including adverse events. The sample represented a clinically severe inpatient population and included patients with a longer-standing course of illness as well as those receiving polypharmacy and requiring behavioral restrictions, thereby reflecting real-world clinical practice.

The treatment continuation rate at week 8 was 53.7%. Previous studies reported continuation rates of 68.1% in a 6-week randomized controlled trial (15) and 47.4% (21) and 38.6% (22) in 52-week extension studies. Baseline severity in those studies was lower than that in our sample, with a mean PANSS total score of 95.2 and CGI-S of 4.9 in the 6-week trial (15), and PANSS scores of 67 with CGI-S values of 3.7 (21) and 4.8 (22) in the long-term trials. Although direct comparison is limited by differences in study design and patient characteristics, the continuation rate in our study appears slightly lower, likely reflecting the inclusion of more severe cases and patients receiving multiple concomitant medications. Recent real-world studies of long-acting injectable antipsychotics (LAIs) have emphasized the clinical importance of treatment continuity in reducing relapse, hospitalization, and emergency service use in schizophrenia-spectrum disorders (23). However, these studies have mainly addressed long-term maintenance strategies, whereas evidence on the short-term continuation and tolerability of oral antipsychotics during acute psychiatric hospitalization remains limited. In this context, our study helps fill this gap by describing the early continuation, tolerability, and reasons for the discontinuation of oral brexpiprazole in acutely hospitalized patients.

Shorter duration since onset, shorter DUP, and lower prior CPZ-eq dose were associated with higher continuation rates, suggesting that earlier intervention may facilitate the therapeutic effects of brexpiprazole regardless of illness severity. Malla et al. reported that brexpiprazole improved both psychiatric symptoms and social functioning in patients within 5 years of onset, highlighting its potential role in early-phase intervention (24). Duration since onset, DUP, and the prior CPZ-eq dose may serve as proxies for underlying illness severity and treatment complexity, and in a retrospective study, residual confounding related to clinician decision-making and concomitant medications cannot be excluded. Therefore, these findings should be interpreted as exploratory and hypothesis-generating, based on unadjusted comparisons, rather than as evidence of independent associations. Given the modest sample size of the current study and the number of unadjusted comparisons in **Table 1**, the possibility of chance findings cannot be excluded, particularly for associations with borderline p values. Accordingly, these baseline associations should be regarded as exploratory and not as evidence of independent predictors of continuation.

Moreover, continuation rates tended to be higher in female patients. A longitudinal study of first-episode schizophrenia found that female patients achieved greater symptom improvement at lower antipsychotic doses and had significantly higher remission and functional recovery rates after 3 years (50% in female patients vs. 30.8% in male patients) (25). In our study, some female patients exhibited manageable psychomotor agitation without treatment modification or discontinuation. Differences in physiological characteristics or staff–patient interactions may have contributed indirectly to the higher continuation rates among female patients, warranting further investigation.

Among patients who discontinued brexpiprazole, the most frequent reason was adverse events (32.3%). Previous studies reported adverse event rates of 48.9–56.7% and 60.4% (15, 21). Since our study recorded adverse events only when they led to discontinuation, direct comparison was not possible. However, only one case of fatigue was related to sedation, consistent with previous reports of the low sedative ability of brexpiprazole (10, 15, 21, 26–28). Akathisia was the most common adverse event observed in our study. Correll et al. and Forbes et al. reported akathisia rates of 4.4% and 4.8% (15, 21), respectively. Although direct comparison is not possible, a higher frequency in our study may reflect the inclusion of patients with more severe illness and greater use of concomitant medication. The second most common reason for discontinuation was insufficient efficacy (29.0%), and all nine patients discontinued within 4 weeks. Ishigooka et al. reported that PANSS total scores improved significantly compared with placebo after 3 weeks of brexpiprazole 2 mg/day (16). Based on this finding, some early discontinuations in our study could be avoided if treatment were continued for slightly longer with appropriate adjunctive medication. Nevertheless, discontinuation decisions in routine acute-care practice are often multifactorial, and assigning a single primary reason from retrospective electronic medical records may not fully capture the overlap between categories (e.g., patient preference/refusal driven by perceived insufficient benefit); therefore, the distribution of discontinuation reasons should be interpreted cautiously as potentially subject to misclassification.

Regarding symptom trajectories, CGI-S and BPRS total scores were lower at 4 and 8 weeks than at baseline among patients who were able to continue brexpiprazole. In BPRS item scores, baseline “Anxiety” scores were higher in the continuation group than in the discontinuation group. The severity of anxiety may have influenced treatment selection (e.g., decisions to continue the index antipsychotic and/or to introduce concomitant medications), and thus residual confounding related to clinical decision-making cannot be excluded. In addition, CGI-S and BPRS are clinician-rated measures and ratings were not blinded; therefore, observer bias cannot be ruled out. Accordingly, these findings should be interpreted as secondary and exploratory. Because there was no control group and the analysis was limited to patients who were able to continue treatment, the observed score reductions should be interpreted as expected clinical improvement among patients who remained on treatment, rather than as evidence of a treatment effect. Regression to the mean also cannot be excluded. Because concomitant psychotropic medication use likely reflected clinical indication, illness severity, treatment complexity, and clinician decision-making, the observed subgroup differences are vulnerable to confounding by indication and should not be interpreted as evidence that concomitant medications themselves influenced symptom trajectories.

This study has some limitations. First, it was a retrospective, single-center study based on clinical records, which may have introduced potential information bias and incomplete data. In addition, 12 patients were excluded before analysis because of insufficient baseline information, death, transfer to another facility, discharge within 8 weeks, or adjunctive use of brexpiprazole. These exclusions, particularly those related to insufficient baseline data and discharge within 8 weeks, may have influenced the composition of the cohort and the observed continuation rate. Second, the 8-week observation period limited the assessment of long-term efficacy, safety, and adherence. In addition, because treatment continuation was assessed during hospitalization, it may have been influenced by tolerability and clinical response as well as by discharge decisions and other institutional factors. Thus, the observed continuation rate may be interpreted as one indicator of short-term inpatient persistence under routine clinical conditions rather than general medication adherence. Third, the brexpiprazole dosage in this study is another limitation. The median dose was 2 mg/day, which is consistent with the recommended dose in Japan, whereas the recommended target dose for schizophrenia in the USA is 2–4 mg/day, with a maximum of 4 mg/day. Accordingly, the present findings may have limited generalizability to international settings where higher maintenance doses are more commonly used. Fourth, the index date (week 0) was not uniform; the study comprised patients who were maintained on brexpiprazole before admission, patients who started brexpiprazole at admission, and patients who were switched from other antipsychotics to brexpiprazole during hospitalization. These clinically distinct treatment-status groups were analyzed together, possibly introducing heterogeneity in tolerability and discontinuation risk. Fifth, many patients received multiple psychotropic agents, and the type and timing of concomitant medications were not controlled, making it difficult to isolate the effects of brexpiprazole monotherapy. Additional interpretive limitations, including residual confounding (e.g., by severity, clinical decision-making, and concomitant medications), potential misclassification of discontinuation reasons, and selection/observer biases, are discussed above. Incorporating more objective measures would strengthen future studies. Prospective multicenter investigations are needed to evaluate the long-term clinical utility of brexpiprazole, clarify its optimal indications, and identify the factors influencing treatment continuation in real-world settings.

In conclusion, this study is one of the first real-world investigations conducted at a university hospital in Japan providing psychiatric emergency care. The study retrospectively described the 8-week brexpiprazole continuation rate (53.7%) and reasons for discontinuation among patients hospitalized with acute schizophrenia. Discontinuation was mainly attributable to adverse events and insufficient efficacy, and the management of adverse events—particularly akathisia—may be important from the standpoint of treatment continuation. The identification of factors associated with continuation (shorter duration since onset, shorter duration of untreated psychosis, lower prior CPZ-eq dose, and female sex) should be considered exploratory. Likewise, symptom trajectories on clinician-rated scales (CGI-S/BPRS) represent secondary, hypothesis-generating findings. Prospective studies are warranted to systematically evaluate treatment continuation and safety in real-world acute-care settings.

## Data Availability

The datasets presented in this article are not readily available because the datasets are not publicly available because they contain potentially identifiable clinical information. Access may be provided upon reasonable request to the corresponding author, subject to approval by the ethics committee and the institution. Requests to access the datasets should be directed to Yuki Noriyama at nori583@naramed-u.ac.jp. Access may be granted upon reasonable request and subject to approval by the ethics committee and the institution.
