# Supplementary material for: Real-world use of brexpiprazole during inpatient treatment for schizophrenia: continuation, discontinuation, and concomitant psychotropics
Source: Front Psychiatry. 2026 May 5;17:1829496. doi: 10.3389/fpsyt.2026.1829496 (PMC13184560; doi:10.3389/fpsyt.2026.1829496)
Supplement: Supplementary file 1 [file Table1.docx]

Supplementary Material

# Supplementary Data

**Table S1. Comparison of baseline BPRS item scores between the brexpiprazole continuation and brexpiprazole discontinuation groups**

| **No.** | **Baseline BPRS item** | **Brexpiplazole continuation group**  **(N=36, median [IQR])** | **Brexpiplazole**  **discontinuation group**  **(N=31, median [IQR])** | **p-value** |
| --- | --- | --- | --- | --- |
| 1 | Somatic Concern | 2.0 (1.3–3.0) | 2.0 (2.0–3.0) | 0.365 |
| 2 | Anxiety | 5.0 (4.0–6.0) | 4.0 (3.0–5.0) | 0.044* |
| 3 | Emotional Withdrawal | 2.5 (2.0–4.0) | 3.0 (2.0–4.0) | 0.633 |
| 4 | Conceptual Disorganization | 5.0 (4.0–6.0) | 5.0 (4.0–6.0) | 0.857 |
| 5 | Guilt Feelings | 2.0 (2.0–3.0) | 2.0 (2.0–3.0) | 0.854 |
| 6 | Tension | 2.0 (2.0–4.0) | 3.0 (2.0–4.0) | 0.995 |
| 7 | Mannerisms and Posturing | 2.0 (1.0–3.8) | 2.0 (1.0–3.0) | 0.411 |
| 8 | Grandiosity | 2.0 (1.0–3.0) | 2.0 (1.0–3.0) | 0.956 |
| 9 | Depressive Mood | 3.0 (2.0–4.0) | 3.0 (2.0–3.0) | 0.597 |
| 10 | Hostility | 3.0 (2.0–5.0) | 3.0 (2.0–4.0) | 0.901 |
| 11 | Suspiciousness | 4.0 (3.0–5.0) | 4.0 (3.0–5.0) | 0.141 |
| 12 | Hallucinatory Behavior | 5.0 (5.0–6.0) | 5.0 (4.0–6.0) | 0.883 |
| 13 | Motor Retardation | 2.0 (2.0–3.0) | 2.0 (1.0–3.0) | 0.343 |
| 14 | Uncooperativeness | 4.0 (3.0–5.0) | 3.0 (3.0–4.0) | 0.373 |
| 15 | Unusual Thought Content | 5.0 (4.3–6.0) | 5.0 (4.0–6.0) | 0.664 |
| 16 | Blunted Affect | 2.0 (2.0–3.8) | 2.0 (2.0–4.0) | 0.926 |
| 17 | Excitement | 3.0 (2.0–5.0) | 3.0 (2.0–5.0) | 0.655 |
| 18 | Disorientation | 1.0 (1.0–2.0) | 1.0 (1.0–2.0) | 0.614 |

Values are presented as median (interquartile range). Comparisons between the brexpiprazole continuation and brexpiprazole discontinuation groups were performed using the Mann–Whitney U test, with results reported as p-values.

Abbreviations: BPRS, Brief Psychiatric Rating Scale; IQR, interquartile range; *p<0.05.

**Table S2. Concomitant psychotropic medications in the brexpiprazole-continuation group**

| **Drug class** | **Medication** | **N** |
| --- | --- | --- |
| Antipsychotics | Olanzapine | 5 |
|  | Aripiprazole | 2 |
|  | Asenapine | 2 |
|  | Quetiapine | 2 |
|  | Risperidone | 2 |
|  | Blonanserin | 1 |
|  | Paliperidone | 1 |
|  | Lurasidone | 1 |
| Benzodiazepines | Bromazepam | 7 |
|  | Clonazepam | 2 |
|  | Diazepam | 2 |
|  | Etizolam | 1 |
|  | Lorazepam | 1 |
|  | Ethyl loflazepate | 1 |
| Mood stabilizers | Valproic acid | 2 |
|  | Carbamazepine | 1 |

Data are presented as n. Because some patients received more than one concomitant medication, counts were not mutually exclusive.
